# Using formalin embalmed cadavers to teach fracture identification with ultrasound

**DOI:** 10.1186/s12909-020-02148-8

**Published:** 2020-07-18

**Authors:** Michael Weston, Dallin Elmer, Scott McIntosh, Nena Lundgreen Mason

**Affiliations:** Rocky Vista University College of Osteopathic Medicine - Southern Utah, 255 East Center Street, Ivins, UT 84738 USA

**Keywords:** Ultrasound education, Fracture identification, Ultrasound in cadavers, Medical education

## Abstract

**Background:**

Ultrasound is being utilized more frequently to diagnose fractures in bone and track fracture reduction quickly, and without radiation exposure in the ED. Realistic and practical methods of teaching sonographic fracture identification to medical trainees are needed. The objective of this study is to determine the feasibility of using formalin-embalmed human cadavers in teaching medical trainees to use ultrasound to identify synthetic fractures in tibia, radius, and metacarpal bones.

**Methods:**

First-year medical students attended an orientation presentation and a 15-min scanning workshop, to evaluate fractures in cadaver bones with an instructor. Next participants independently scanned bones to determine if a fracture was present. Questionnaires were given that assessed participant self-confidence and ability to evaluate still ultrasound images for fracture and differentiate between tissue layers before, after, and 5 months following training.

**Results:**

Participants were collectively able to scan and differentiate between fractured and unfractured bone in 75% of 186 total bone scanning attempts (tibia: 81% correct, metacarpal: 68% correct, radius: 76% correct). When evaluating still ultrasound images for fracture, participants’ scores rose significantly following training from an average score of 77.4 to 91.1% (*p* = 0.001). Five months post-training, scores fell slightly, to an average of 89.8% (*p* = 0.325).

**Conclusions:**

Ultrasound images of formalin-embalmed cadaveric fractures are of sufficient quality to use in teaching fracture identification to medical trainees. With only 15 minutes of scanning experience, medical trainees can learn to independently scan and significantly increase their ability to identify fractures in still ultrasound images.

## Background

Ultrasound technology is becoming immensely popular in many medical disciplines due to its, modest cost, rapid imaging, and low risk to patients, when compared with other imaging modalities [1, 2]. These essential benefits have motivated the implementation of ultrasound into a wide array of medical specialties ranging from primary care to emergency medicine, as well as for bedside use and prehospital procedures [3–5]. Clinically, ultrasound has been shown to be a useful diagnostic tool to accurately identify fractures in many types of bones such as the ribs, radius, metacarpal, and tibia [3–7]. On average, by utilizing ultrasound in the diagnosis of long bone fractures, the overall time to diagnosis can be reduced to an average of 4 minutes by performing bedside evaluation [8]. Ultrasound has also proven to be useful in detecting occult fractures not readily visible with normal radiography [9]. Additionally, Ultrasound has been shown to have great utility in the stepwise evaluation of fractures during fracture reduction in the ED across many bone types [10, 11].

As new clinical applications are developed, practical methods to teach medical trainees ultrasound skill are needed [12]. Early integration of ultrasound education into medical training incorporates a key technology which will better equip students to become modern physicians [13]. Studies have shown that many types of learners can perform ultrasound to identify fractures [3, 12, 14]. Medical students have also successfully used ultrasound to identify fractures in chicken bones [14]. Because many undergraduate medical education programs have access to cadaver laboratories, formalin-embalmed human donors may provide a practical solution to incorporate ultrasound fracture identification into medical training early on. Introducing this type of training to medical students in a low-stress pre-clinical setting such as an anatomy lab, allows the learning of a valuable clinical skill without the time constraints, potential for patient discomfort, or poor patient compliance, that they would encounter learning these skills in a clinical setting for the first time [15]. Despite the apparent benefits, there is much skepticism within the medical and scientific communities regarding the utility of formalin-embalmed cadavers in ultrasound training. The general lack of water permeated throughout formalin-embalmed tissue tends to yield ultrasound images of poor quality when compared to fresh-frozen or Thiel (soft) embalmed cadavers [16–18]. However, formalin embalming is the most common method of preservation utilized to prepare donors for use in teaching gross anatomy to medical students, making them a more readily avalable resource for most medical programs [19].

## Methods

### Study overview and aims

The goal of this study was to determine the feasibility of using formalin-embalmed human cadavers in teaching medical trainees to use ultrasound to identify synthetic fractures in tibia, radius, and metacarpal bones. In order to fully meet this objective, two aims need to be addressed. First, it must be determined if synthetic fractures could be created in cadaver bones that are clearly visible with ultrasound imaging. Ultrasound images of several fractures are presented in this work to demonstrate that the quality of imaging is sufficient for use in fracture identification training. Secondly, the utility of the formalin-embalmed cadaver model in teaching novice medical trainees to identify fractures must be explored. A training workshop was conducted allowing first-year medical students who were inexperienced sonographers, to use ultrasound to look for synthetic fractures and be assessed on their accuracy. Study participants were also given questionnaires that assessed knowledge and self-confidence before, after, and 5-months following training. Because medical programs that have access to ultrasound also likely have access to cadavers, the use of synthetic cadaveric fractures could be incorporated into any curricula as a practical, low risk, and low-stress, way to introduce ultrasound fracture identification to medical trainees early on in medical education.

### Ethics and approval

This study was reviewed and determined to be exempt by the institutional review board (IRB) of Rocky Vista University prior to beginning any research-related activities (IRB #20180018). All participants signed an IRB approved written consent form before the study began. Permission to use human cadavers in this project was obtained from the University of Utah Body Donor Program.

### Equipment

Six Echo 5 ultrasound machines, equipped with 5-10 MHz linear transducers (Chison Medical Technologies Co. Ltd., Jiangsu, China) were used at teaching stations. At testing stations, there were Mindray Z5 portable ultrasound machines each equipped with a 5-10 MHz linear transducer (Mindray Medical International Ltd., Shenzhen, China). Ballistics gel (Clear Ballistics LLC, Greenville SC) and transducer gel (McKesson Medical-Surgical Inc., Richmond, VA) were also utilized. Questionnaires were administered using Google Forms (Google LLC, Mountain View, CA).

### Study setting and population

This study was conducted at an accredited college of osteopathic medicine in the United States. The opportunity to participate in this study was advertised by mass email and common area postings to all 145 first-year medical students. Thirty-one first-year medical student volunteers without any prior training in musculoskeletal ultrasound were enrolled as participants. No student-volunteers were excluded from participating. First-year medical students were selected for inclusion in this study over upperclassmen, due to their inexperience in the interpretation of ultrasound images and operation of ultrasound equipment. Thirty of the 31 participants reported that they had never received any training covering sonographic fracture identification prior to participation in this study. All participants reported they had less than 5 hours prior experience using an ultrasound machine.

### Aim 1: fracture creation and evaluation

**Fig. 1:**
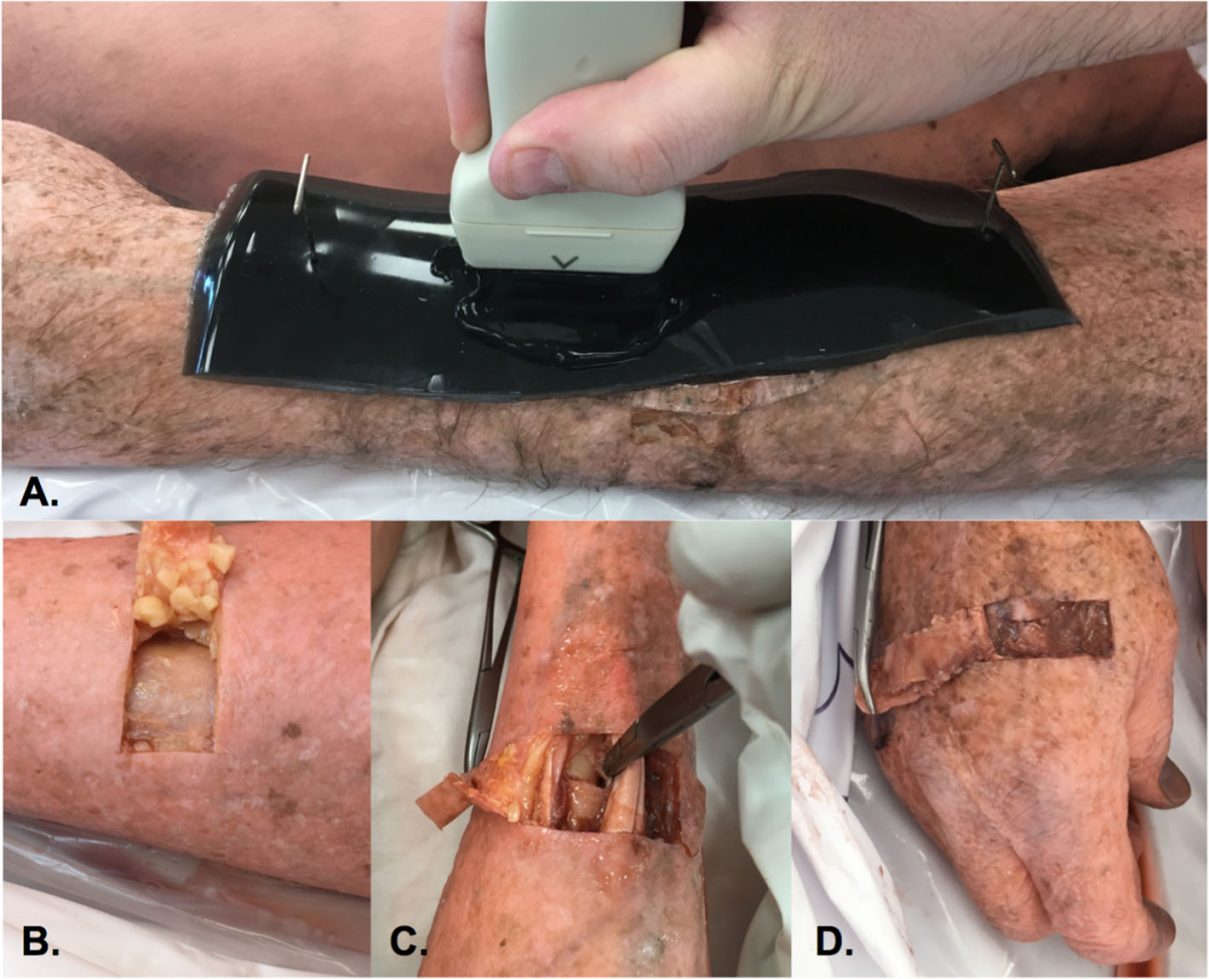
Synthetic Fracture Creation. A series of images depicting how synthetic fractures were created and scanned in cadaver bones. Panel **a** shows how step-off pads and steel T-pins were used to clearly demarcate scanning sites. Panel **d** depicts the formation of a skin/tissue flap over a cadaveric tibia. Panels C and D depict synthetic fractures in the radius and 2nd metacarpal respectively

In order to determine if synthetic fractures can be created in cadavers that are clearly visible with ultrasound imaging, fractures were created in cadaver bones by first using a scalpel to elevate a narrow flap of skin and tissue to expose each target bone. (Fig. 1 panel B). Next an ostetome and mallet were used to create a series of fractures in several types of bones (Fig. 1 panels C-D). Only a single fracture was created in each bone. Care was used to insure each fracture ran cleanly through the entire circumference of each bone without causing it to completely shatter into many pieces, or result in excessive tearing of surrounding structures.

Some fractures were made transversely across the bone as shown in Fig. 1, while others were made at angles to the bone’s length. In creating a skin flap and fracture, air is introduced into the tissue between the skin and bone that makes ultrasound imaging difficult. To solve this problem, following fracture, a small amount of transducer gel was placed between the bone and the skin flap, to insure adequate sound transduction to the bone. A ballistics gel step-off pad (6″ × 3″ × 1/2″) was placed on top of each fracture/pseudofracture site and secured to the cadaver with steel T-pins (Fig. 1 panel A).

Each synthetic fracture was then imaged by an Associate Professor of ultrasound employed by the college of medicine to confirm that each fracture yielded ultrasound images of high enough quality to be used in teaching fracture identification to medical trainees. Criteria of a satisfactory fracture included a sonographically visible step-off sign that varied less than 0.5 cm from the periosteal line, and sufficient gel placement to insure adequate sound transduction to the fracture site. A half centimeter was utilized as the cut-off point for periosteal displacement to insure the step-off sign in each fracture was not overly obvious, and to insure each fracture would be undetectable upon visual inspection along the length of the bone.

Participants were instructed to only scan the region between the T-pins. This decreased the time that participants needed to search for fracture sites, since a limited amount of time was allowed to complete each scan. Step-off pads at each teaching station were translucent, and pads at testing stations were dyed black. These pads served several functions; they ensured that the skin flap was not displaced during scanning, protected the transducer from chemical contamination from the cadaver, and prevented the participants from visually locating the fracture site. Each fracture was imaged with ultrasound to insure the sonographic step-off sign was just large enough in magnitude to be visible to a novice sonographer, but was not overly obvious.

### Aim 2: training activities and assessments

To evaluate the utility of a cadaver model in teaching novice medical trainees to identify fractures using ultrasound participants first attended a 30-minute orientation PowerPoint presentation (Microsoft Corporation, Redmond, Washington, USA). The presentation covered the basics of ultrasound terminology, gain and depth functionality, and how to maneuver through the scanning stations in the ultrasound workshop. The presentation also covered the sonographic appearance of fractured bone, and joint spaces in human cadavers. Following the orientation presentation, participants attended a hands-on scanning workshop. The workshop contained a total of 6 cadaver stations, three for teaching and three for testing. Cadaver stations were grouped by the specific type of bone being evaluated for fracture (tibia, metacarpal, or radius).

### Cadaver stations

Cadavers at each of three teaching stations had bilateral fractures in either the radius, tibia or metacarpal bones. This gave participants an opportunity to scan two fractured bones of the same type at each teaching station. With the aid of an instructor, participants were given 2 minutes to practice scanning each side of each teaching station cadaver. Three testing station cadavers were also prepared by creating fractures on one side of each cadaver in either a radius, tibia or metacarpal bone. At each testing station the left or right side was randomly selected for fracture. On the contralateral side, the fracture creation procedure was reproduced excluding the actual bone fracture step, to create an unfractured pseudofracture site. Participants were given 1 minute to independently scan each side of the cadaver at each testing station and determine if a fracture was present. In total, participants spent 15 min learning to scan at the teaching stations with an instructor, and 6 min scanning independently to determine if fractures were present at the three testing stations.

### Assessments: scanning workshop

During the scanning workshop, each testing station was equipped with paper response forms. Participants used these forms to check boxes to indicate whether they thought each bone they scanned was fractured or unfractured.

### Assessments: questionnaires

Participants were given pre- and post-training digital questionnaires which asked them to assess still ultrasound images for fracture, and distinguish between the sonographic appearance of relevant tissue layers. The questionnaires also assessed each participant’s ability to differentiate between the appearance of a fracture and a joint space on ultrasound imaging, since novice sonographers frequently mistake one for the other. The questionnaire was also sent to participants 5 months following training to assess their retention. All questionnaires contained the same questions with one exception. The post-training questionnaire ended with an optional section allowing participants to submit comments they had about their experiences in the study. All questionnaires were accessed digitally using a Google-form. Pre- and 5-months post-training questionnaires were sent to participants via email. The post-training questionnaire Google-form, was accessed by participants directly following the workshop on provided laptop computers.

### Statistical analysis

**Fig. 2:**
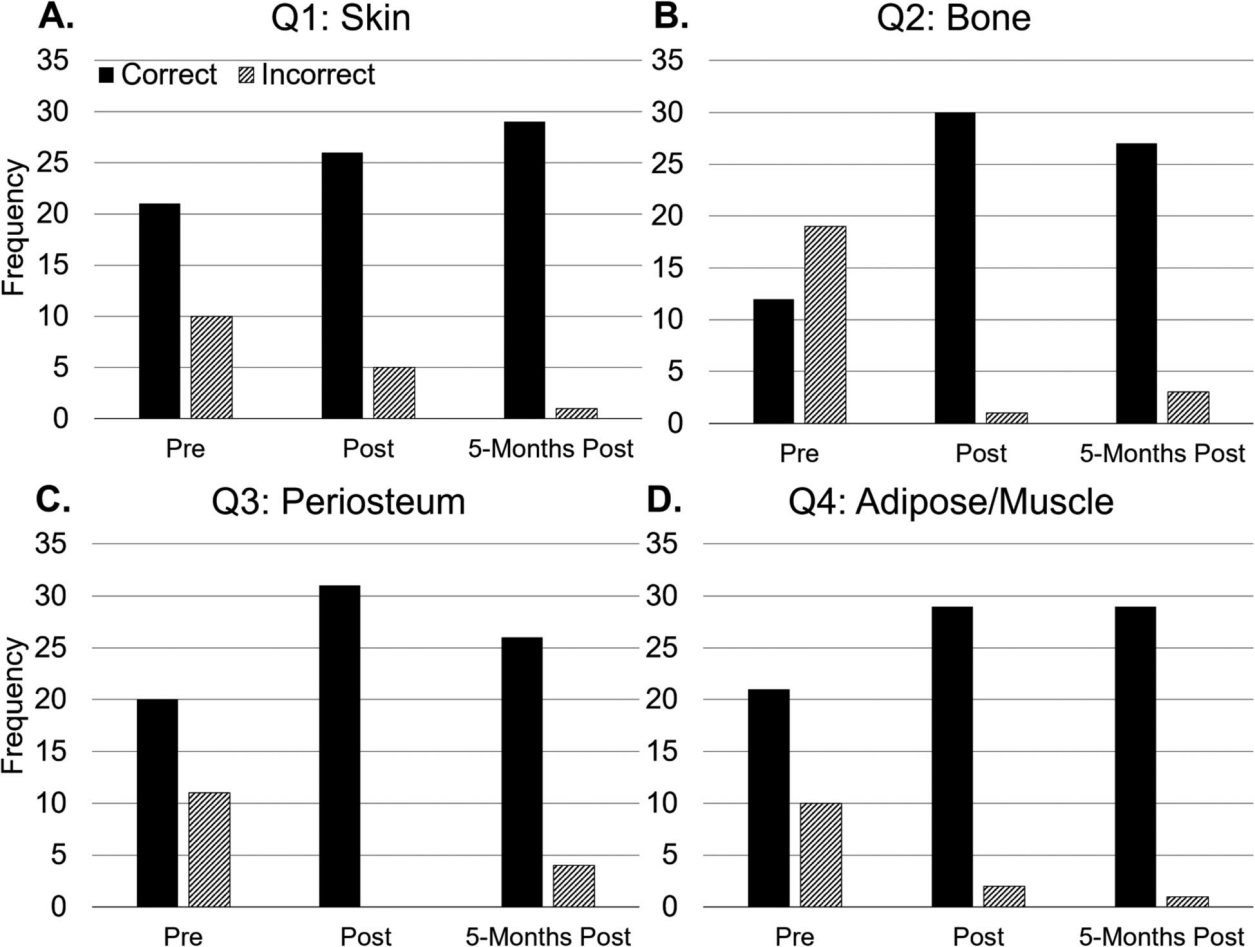
Differentiation Between Tissue Layers. A series of bar graphs depicting participant responses to a group of questionnaire items that asked participants to identify the sonographic appearance of relevant layers of tissue as seen on still ultrasound images of cadaveric bone. The Y axis of each panel represents the number of participant responses, and the X axis indicates questionnaire items. Panels **a-d** display the number of correct (black bar) versus incorrect (shaded bar) responses. Analysis via Chi-squared test yielded the following *p*-values Q1(*p* = 0.302), Q2(*p* = 0.001), Q3(*p* = 0.001), Q4(*p* = 0.021) from pre- to post-training. From post- to 5-months post-training, the *P* values were calculated to be Q1(*p* = 0.093), Q2(*p* = 0.285), Q3(*p* = 0.036), and Q4 (*p* = 0.575)

Prior to analysis participant data was de-identified by replacing each participant name with a randomly generated alphanumeric code. Data analysis was performed by a third-party statistician who had no conflict of interest with the research team. Analysis was completed using SPSS 25 (International Business Machines Corporation, Armonk, New York, USA), and Excel 365 (Microsoft Corporation, Redmond, Washington, USA). Pre and post-training responses were analyzed using a one-tailed T-test after the data was determined to be normally distributed. A Chi-square test was used to the analyze pre- and post-training responses seen in Fig. 2. A standard alpha of 0.05 was utilized to determine statistical significance.

## Results

### Aim 1: fracture creation and evaluation

The first aim of this study was to determine if synthetic fractures could be created in cadaver bones that were clearly visible with ultrasound imaging. Figure 4 depicts the quality of ultrasound images acquired of cadaveric fractures at each testing station, and each station’s accompanying participant score data. In the top row, panels A2-A3 show the ultrasound imaging quality of the fractured and unfractured sides of the tibia station. Panel A1 shows the scores of participants when labeling each side of the tibia testing station as fractured or unfractured. This same panel organization is conserved when viewing rows B and C of Fig. 4 which depict the data and ultrasound images from the metacarpal and radius stations respectively. Overall the participants were collectively accurate in scanning and labeling bone as fractured or unfractured in 75% of 186 bone scanning attempts. (tibia: 81% correct, metacarpal: 68% correct, radius: 76% correct).

### Aim 2: training activities and assessments

**Fig. 3:**
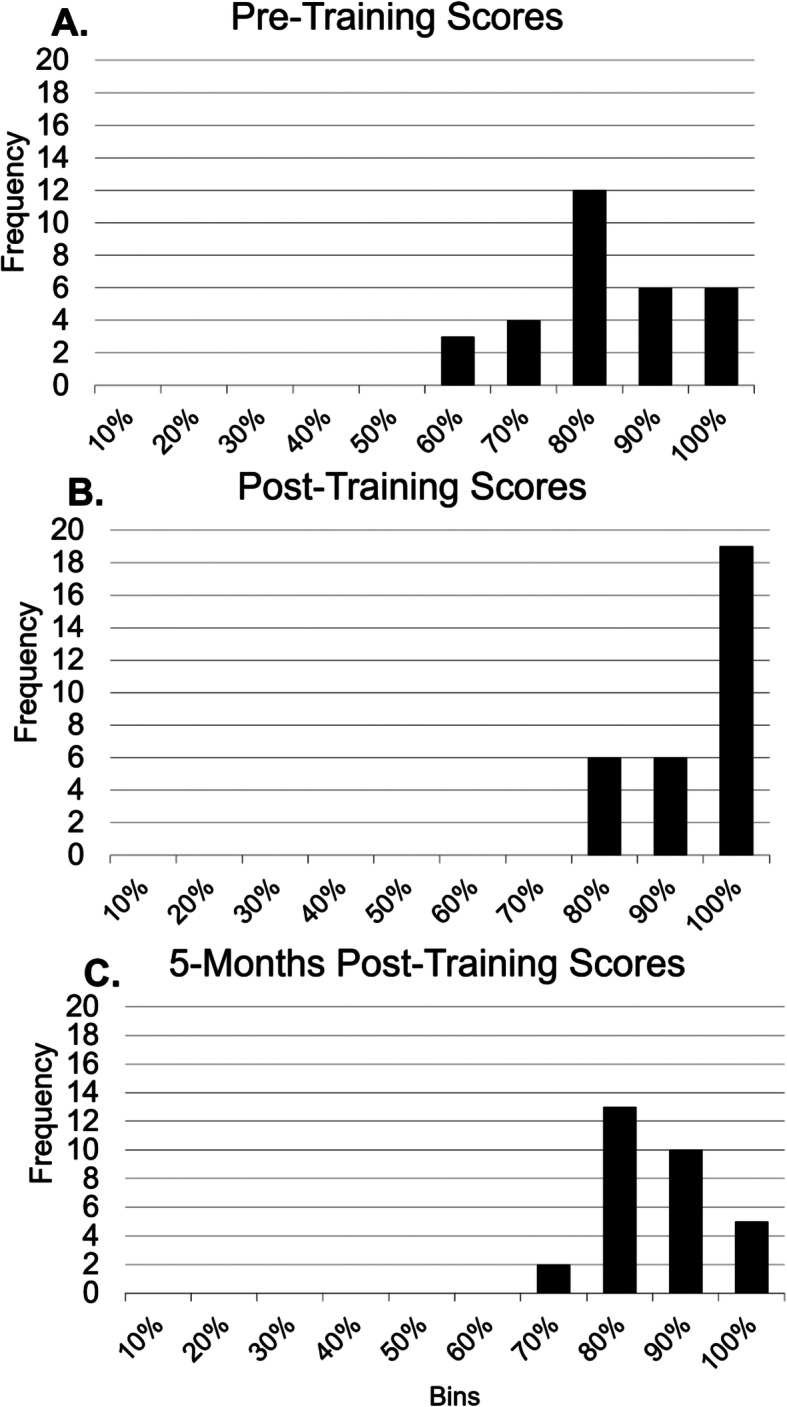
Identification of Fractures on Still Ultrasound Images. An array of bar graphs depicting participant scores on a series of questionnaire items that asked participants to evaluate still ultrasound images of cadaveric bones for fracture. Participant scores were analyzed via a one-tailed T-test and then aggregated into bins spanning 10 percentage points. The Y axis of each panel represents the number of participant responses. Panels **A-C** display results of the pre-, post-, and 5-months post-training assessments respectively. The mean score of 77.4% rose to 91.7% following training (*p* = 0.001). Five months post-training, the average score fell slightly to 89.8% correct (*p* = 0.325)

The second aim of this study was to determine the utility of the formalin-embalmed cadaver model in teaching novice medical trainees to identify fractures. A questionnaire was administered that asked participants to distinguish between tissue layers in ultrasound images. Data depicted in Fig. 2 shows a statistically significant increase in correct responses from pre- to post-training in the identification of bone (panel B, *p* = 0.001), periosteum (Panel C, *p* = 0.001), and adipose/muscle (Panel D, *p* = 0.021). There was not a significant increase in correct responses when identifying skin (Panel A, *p* = 0.302). The proportion of correct responses varied only slightly 5 months following training in the identification of skin (Panel A, *p* = 0.093), bone (Panel B, *p* = .285), periosteum (Panel C, *p* = .036), adipose/muscle (Panel D, *p* = 0.575),

Participants were also asked to review still ultrasound images of cadaver bones and determine if each image contained a fracture. Figure 3 shows that there was a significant increase in correct participant responses following training, with a mean score of 77.4% pre-training (panel A) rising to 91.7% post-training (panel B) (*p* = 0.001). Five months post-training the average score fell slightly to 89.8% correct (*p* = 0.325). When participants were asked to distinguish between a joint space and a fracture in a still ultrasound image, there was a significant increase in selection of the correct answer (joint space) after training (*p* = 0.012). There was an insignificant decrease in correct responses between post- and 5-months post-training assessments (*p* = 0.285). These *p* values were calculated via one-tailed T-test.

When invited to submit free-response comments on the post-training questionnaire, a majority (85%) of submitted participant comments were exclusively positive in nature. Participants made many statements like “It was so helpful to learn what the fractures looked like, and especially to be able to find them on my own with ultrasound” and “This was a really great learning experience and a wonderful educational tool”. Eighteen percent of participants also highlighted the short period of time allotted to scanning as a weakness of the experience overall along with their positive comments. For example, the participants submitted comments like “Great experience, a little rushed but fun!” and “I really enjoyed it! I feel like if I was able to go back into the lab without a timer and play around more, I would be more confident” and lastly, “I had a hard time finding the periosteum before the time ran out”.

## Discussion

### Feasibility of visualizing formalin-embalmed cadaver fractures with ultrasound

**Fig. 4:**
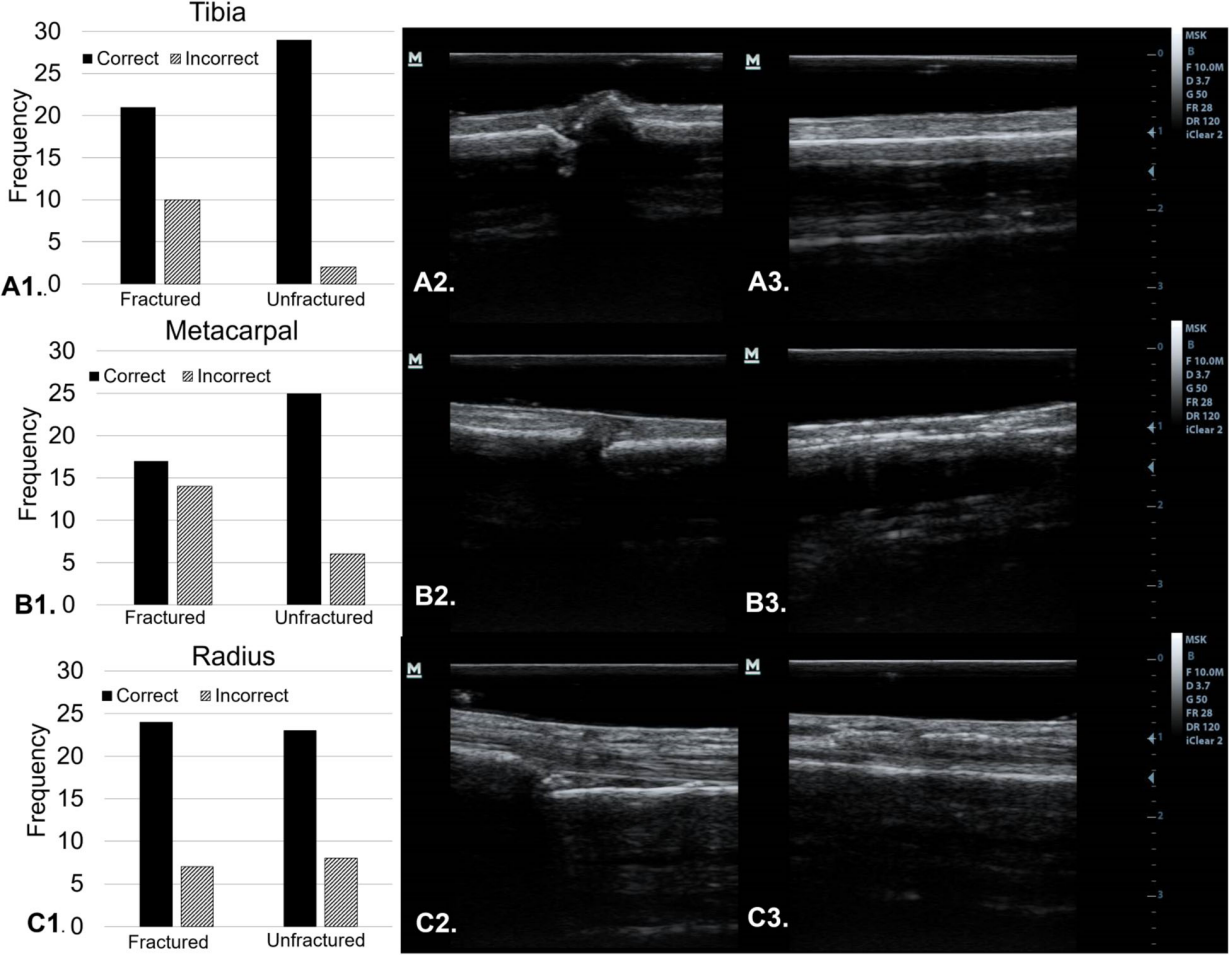
Ultrasound Workshop Testing Stations. Panel A1 depicts participant responses when asked to scan a tibia and determine if it was fractured or unfractured. Panels A2 and A3 show instructor acquired ultrasound images of the fractured and unfractured sides of the tibia testing station cadaver respectively. This same panel organization is conserved when viewing rows B and C of Fig. 4 which depict the data and ultrasound images from the metacarpal and radius stations respectively. The Y axis of panels A1, B1, C1 represent the number of participant responses

Panels A2–3, B2–3, & C2–3 of Fig. 4 show ultrasound images acquired of the synthetic fractures created at each testing station. These images prove that synthetic fractures in formalin-embalmed cadaver bones are clearly visible with ultrasound imaging. In each ultrasound image, the hyperechoic periosteal line, and sonographic step-off sign of each fracture are easily visible, even to novice medical trainees. These novel images qualify the formalin-embalmed cadaver as a viable and useful tool in ultrasound fracture identification training.

### Utility of cadavers in fracture identification training

Figure 2 shows that participants were significantly better-able to correctly distinguish between the sonographic appearance of relevant tissues after training. However, when participants were asked to identify skin (panel A), there was not a significant increase in correct responses from pre- to post-training. This is likely because the pre-training scores were quite high at the onset. Unsurprisingly, this indicates that many participants were aware that skin is the most superficial layer and were able to identify it without training. Participants who missed this question, likely failed to correlate the near-field of an ultrasound image with superficial structures, and the far-field with deeper structures.

When tracking scores in skin identification from post- to 5-months post- training, there was small, but statistically significant jump in correct responses (*p* = 0.093). The 8 hours of curricular ultrasound laboratories (MSK I & II, abdomen & cardiac) that participants attended between these two assessments may explain the increase in correct responses. It is worth noting that these four curricular ultrasound sessions did not contain fracture identification training of any kind, but did allow the participants to gain more experience with sonographic tissue identification. Overall, participants were generally less aware of the sonographic appearance of the deeper tissue layers prior to training, and were able to significantly increase their knowledge regarding the organization of these layers, and their sonographic appearance through participation in this study.

Data depicted in panel A of Fig. 3 shows that medical students who are completely untrained in sonography can be reasonably successful (mean score of 77.4%) in the identification of fractures in still ultrasound images. It is true that a pre training average score of 77.4% is quite high, indicating that even untrained students can usually recognize clear examples of step-off sign. However, there was a significant increase in the scores after training (mean score of 91.7%), indicating that the participants were better-able after training to catch the more-subtle fractures featured in many questionnaire items. Five-months post-training the mean dropped only slightly to an average score of 89.8%, demonstrating that participants had the ability to retain the knowledge needed to recognize fractured bone. The workshop provided participants with only a brief 15 minutes each of scanning time. Before incorporating cadaveric fracture training into our ultrasound curriculum we intend to increase the amount of scanning time available to the students.

The questionnaires also asked participants to recognize a joint space on a still ultrasound image because the sonographic appearance of joints are often mistaken for fractures by novice sonographers. On this question, a significant increase in correct responses was observed after training (*p* = 0.012). Between post- and 5-months post-training, the score fell significantly to 63.3% correct (*p* = 0.285) indicating that the participants were not able to retain this skill as well as others that were assessed in the 5-month follow up questionnaire.

Figure 4, panels A1, B1, & C1 show the data collected following the hands-on scanning workshop. Participants were able to successfully scan independently, and were largely able to correctly identify bones as fractured or unfractured. Overall the participants were collectively accurate in scanning and labeling bone as fractured or unfractured in 75% of 186 bone scanning attempts. The participants seemed to struggle the most with the metacarpal station (panels B1-B3). The authors attribute this to the fact that the sonographic step-off sign at the metacarpal station was the smallest in magnitude when compared to the tibia and radius stations (see panels A2, B2, C2). Additionally, the metacarpal itself, is a smaller bone and was therefore more difficult to image with ultrasound. This conclusion is supported by past studies which indicate that fractures in bones that are larger and longer are easier to identify with ultrasound than smaller or irregularly shaped ones [20].

Data in Fig. 4 (panels A1, B1, C1) suggests that overall participants are more accurate in correctly identifying unfractured bone than fractured bone. The authors believe this could have been influenced by lack of time. Participants were given 1 minute to scan each side of each testing station cadaver and record their answer. They were not given any additional time to transition from one side to the other, which may have caused some participants to be unable to locate the fracture in the allotted time and label the bone as unfractured by default. Data from the radius station (panel C1) did not show this discrepancy, indicating that participants found the radial fracture easiest to distinguish and were able to successfully make their answer selection in the allotted time.

### Limitations

This study is not without limitations. Firstly, there was no control group with which to compare participants’ scores. However, the aim of this study was not to directly compare cadaver-based fracture identification training to other teaching modalities, but instead to determine the feasibility of using formalin-embalmed cadavers in teaching fracture identification. The authors were interested in exploring this possibility because the formalin-embalmed cadaver is often dismissed when being considered for use in ultrasound training due to the poor quality of ultrasound images they generally yield because of lack of water in the tissue.

Additionally, there were a few logistical issues that likely attenuated the success of the participants. Testing station transducers were covered in sterile plastic transducer bags and teaching station transducers were not. The transducer bags were used to protect the testing station transducers from the dye in the ballistics gel step-off pads. The authors suspect that this difference between the training and testing stations, although necessary at the time, may have distracted the participants from being as successful as they could have been. Also, allowing the participants more time to scan and transition between the sides of the cadavers would likely increase participant success. Lastly, synthetic fractures generated for this project were relatively simple fractures which all exhibited visible step-off sign. Real fractures encountered clinically by various practitioners vary greatly in presentation and sonographic appearance. The wide variety of possible fracture types are not well represented in this study, and therefore the direct application of this study to clinical performance of fracture evaluation is somewhat limited. However, participants did gain valuable practice, handling ultrasound equipment, scanning bones, recognizing orthopedic sonographic landmarks, and identifying step-off sign in human bone making this a valuable learning experience.

## Conclusions

Synthetic fractures in formalin-embalmed human cadavers can be used to effectively teach inexperienced medical trainees to identify fractures with ultrasound. The quality of images obtained during this study, are sufficient to utilize in teaching inexperienced learners to identify fractures despite having been acquired using low to mid-level quality ultrasound machines (Fig. 4, panels A2–3, B2–3, & C2–3). This is of particular relevance when considering the difficulty of obtaining usable sonographic images in most regions of formalin-embalmed cadavers, due to a general lack of water permeated throughout formalin-embalmed tissue [16, 17]. Because many medical programs with access to ultrasound machines, also have access to cadaver laboratories, formalin-embalmed cadavers can provide excellent and readily available tools for teaching medical trainees ultrasound-related clinical skills in an educational setting. Teaching medical trainees clinical skills in a low-stress pre-clinical setting offers significant benefits, like freedom from time constraints and the potential for patient discomfort [21]. Data compiled from this study indicates that with a brief orientation presentation, and as little as 15 min of hands-on training scanning fractures in human cadaveric bone, medical trainees can learn to scan, and significantly increase their ability to identify fractures independently.

The use of human cadavers and ultrasound imaging in fracture identification training can have a positive effect on patient care. Trainees can learn without inconveniencing real patients with fractures who are in pain and don’t want to be used in teaching opportunities, while still having the opportunity to scan fractures in real human tissue. If these skills are well taught early on in medical education, point-of-care ultrasound can be utilized to quickly diagnose many types of bony fractures and assist in reducing them quickly and accurately at the bedside. Point-of-care ultrasound can also save time, patient radiation exposure, and drive down health care costs by avoiding radiology in some fracture cases.

## Data Availability

The datasets used and/or analyzed in this study are available from the corresponding author on reasonable request.

## Abbreviations

IRB: Institutional review board
MHz: Megahertz
MSK: Musculoskeletal
